# Solitary Neurocysticercosis: A Challenging Diagnosis, a Simple Treatment

**DOI:** 10.7759/cureus.96293

**Published:** 2025-11-07

**Authors:** Laila M Baydhi, Abdulrahman A Shamakh, Riadh M Rebai, Fahdah K Musallam

**Affiliations:** 1 Neurosurgery, King Abdulaziz Medical City, Ministry of National Guard Health Affairs, Jeddah, SAU; 2 Medicine and Surgery, King Fahad General Hospital, Jeddah, SAU; 3 Neurosurgery, King Fahad General Hospital, Jeddah, SAU

**Keywords:** cns parasitic infection, neurocysticercosis, new-onset seizure, solitary cysticercal granuloma, taenia solium

## Abstract

Neurocysticercosis (NCC), caused by the larval stage of *Taenia solium*, is the most common parasitic infection of the central nervous system and a major cause of adult-onset epilepsy in endemic regions. While typically presenting with multiple brain lesions, solitary parenchymal lesions can occur and may mimic tumors, abscesses, or granulomatous diseases, especially in non-endemic settings where clinician familiarity is low.

We report the case of a young Filipino woman living in Saudi Arabia who presented with new-onset seizures and a solitary parietal lesion. Limited epidemiologic risk factors and atypical imaging findings delayed diagnosis until histopathology confirmed NCC. This case underscores the importance of including NCC in the differential diagnosis of solitary brain lesions even in non-endemic regions.

## Introduction

Neurocysticercosis (NCC) results from infection of the central nervous system by the larval stage of *Taenia solium*. It is a major cause of adult-onset epilepsy in endemic regions such as Latin America, sub-Saharan Africa, and parts of Asia, including the Philippines [1]. Clinical manifestations vary widely, with seizures being the most common presentation. Parenchymal disease often produces multiple lesions, but solitary lesions may also occur, posing diagnostic challenges. In non-endemic countries, such as those in the Middle East, limited awareness can lead to misinterpretation of solitary cystic lesions as neoplasms, abscesses, or other granulomatous diseases. Histopathological confirmation remains essential when imaging and laboratory results are inconclusive. We describe a case of solitary NCC in a young woman with no known exposure history, highlighting the diagnostic difficulties in non-endemic areas.

## Case presentation

A 29-year-old Filipino woman residing in Saudi Arabia, with no significant past medical history, had no history of fever, cough, contact with tuberculosis patients, family history of similar illnesses, or routine medication use. She presented to the emergency department following a first episode of generalized tonic-clonic seizure. There was no personal or family history of epilepsy or neurological disorders. On initial examination, she was afebrile, alert, and oriented. Her general physical examination was unremarkable, with no signs of lymphadenopathy. Neurological examination showed no focal deficits.

**Figure 1:**
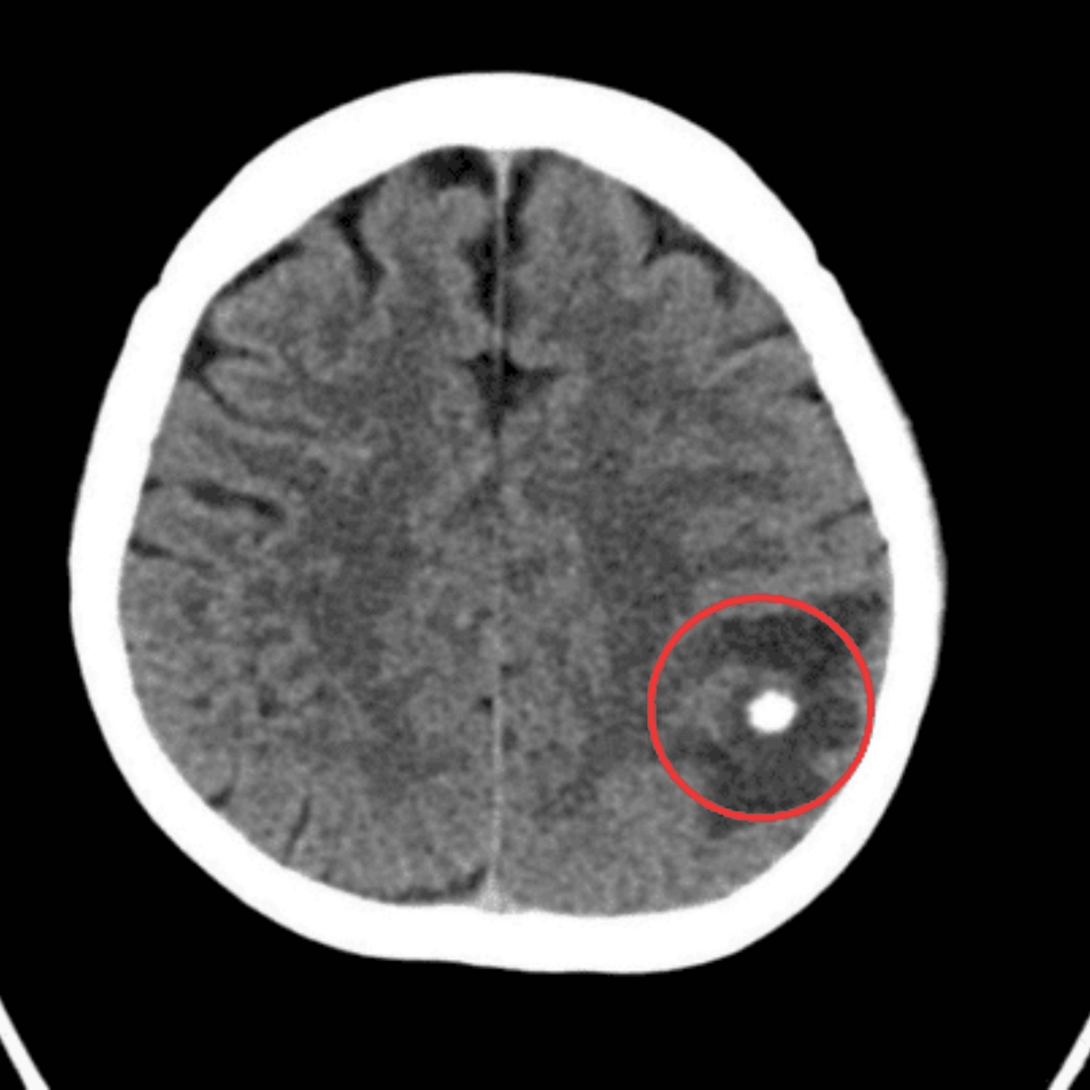
Non-contrast CT brain showing a hyperdense lesion in the left parietal lobe (red circle) with minimal surrounding edema, likely representing a calcified granuloma.

A non-contrast computed tomography scan of the brain revealed a focal hyperdense lesion in the left parietal lobe, raising suspicion for a calcified mass or granuloma (Figure 1). The patient was started on levetiracetam for seizure control. During hospitalization, she remained clinically stable and asymptomatic. Laboratory investigations, including complete blood count, inflammatory markers (CRP and ESR), and tuberculosis screening, were within normal limits. Serologic tests for NCC and other parasitic infections were negative.

**Figure 2:**
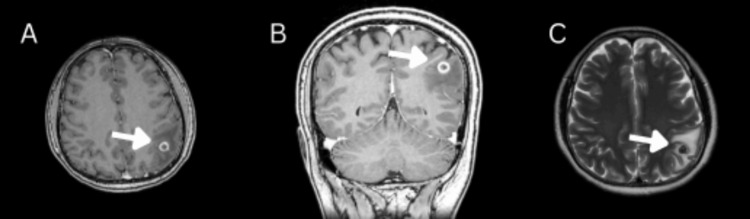
MRI brain showing a ring-enhancing lesion in the left parietal lobe with surrounding edema, as indicated by the white arrow. (A) Axial T1-weighted post-contrast image, (B) coronal T1-weighted post-contrast image, and (C) axial T2-weighted image. The lesion demonstrates a hypointense center with an enhancing rim on T1 post-contrast sequences and surrounding hyperintense edema on T2-weighted imaging, consistent with a calcified granuloma.

Further imaging with contrast-enhanced magnetic resonance imaging (Figure 2) demonstrated a 1 cm ring-enhancing lesion in the left parietal lobe, with mild perilesional edema but no midline shift. Due to the solitary nature of the lesion, negative serologies, and diagnostic uncertainty, a decision was made to proceed with surgical intervention for definitive diagnosis and treatment.

**Figure 3:**
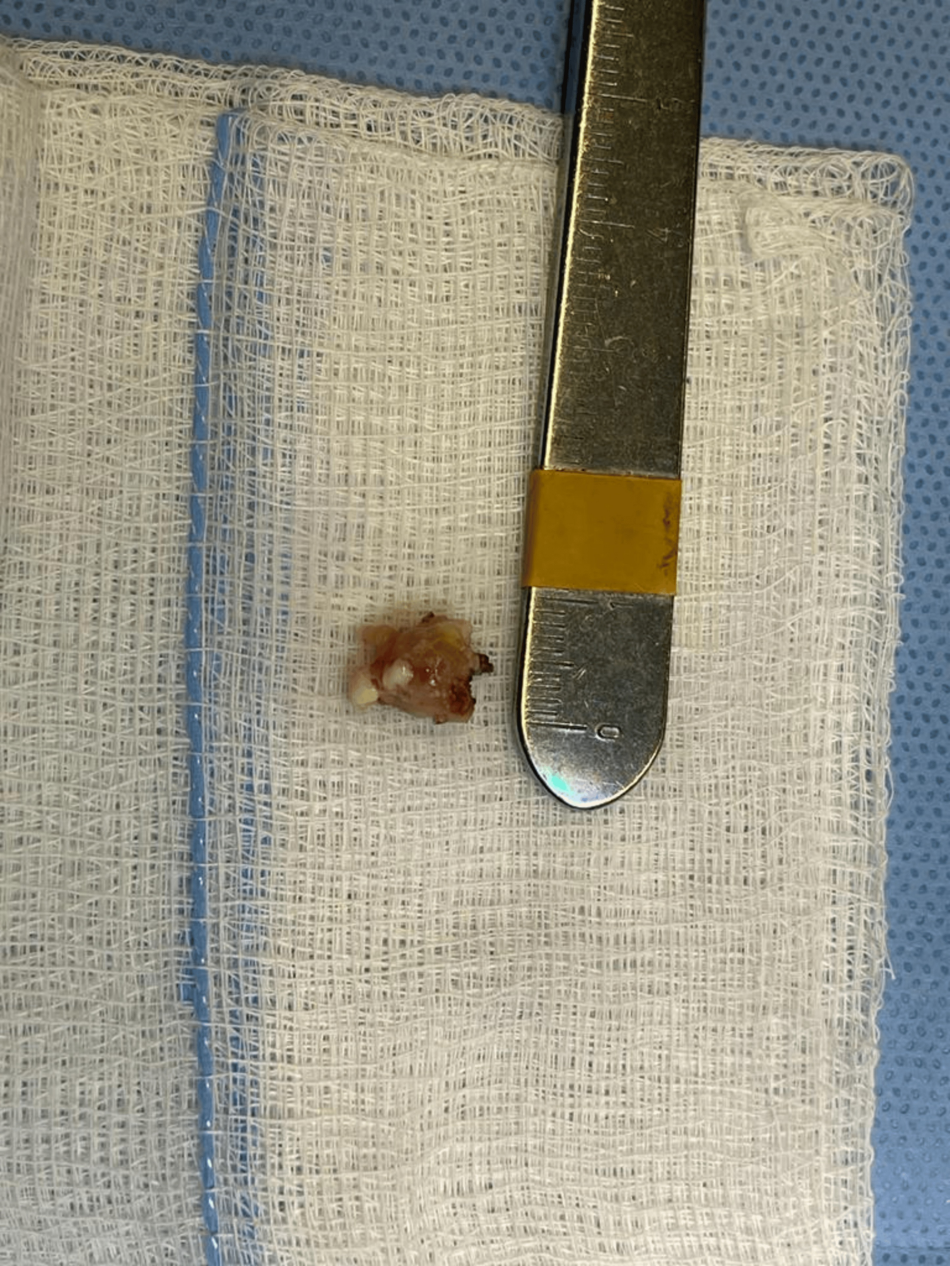
The gross specimen demonstrated a 1 cm calcified cyst

A neuronavigation-guided left parietal craniotomy was performed using a trans-sulcal approach. Intraoperatively, a firm, calcified mass was identified and excised completely without complications (Figure 3).

**Figure 4:**
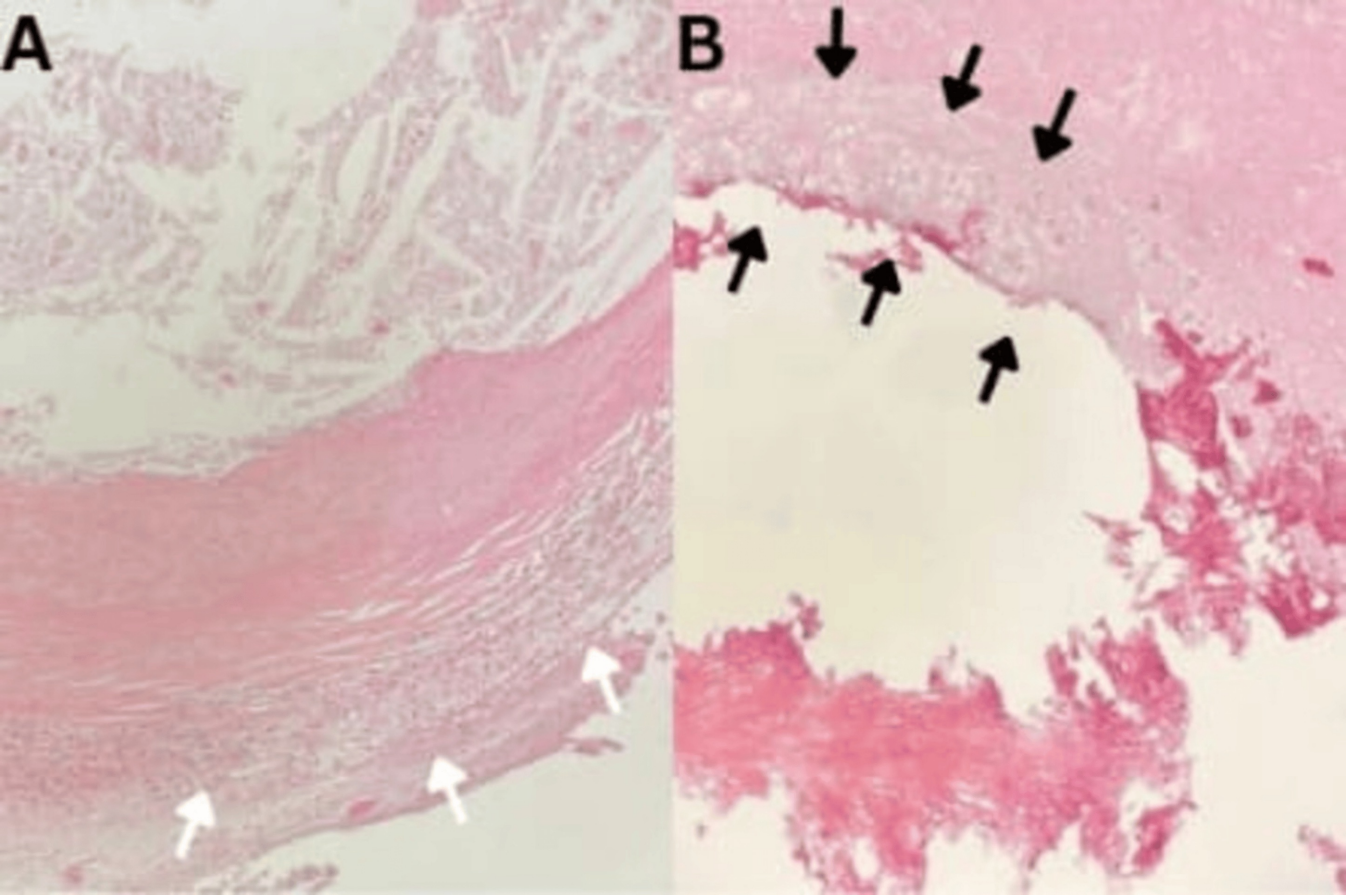
Microscopic examination revealed granular debris and a surrounding chronic inflammatory infiltrate (indicated by white arrows in image A). The lesion was well-demarcated by a fibrous wall that lacked an epithelial lining (indicated by black arrows in image B). These features are consistent with a degenerating and calcified cysticercus, which is a characteristic of neurocysticercosis in the late granular-nodular to early nodular-calcified stage.

Histopathological examination revealed a well-circumscribed lesion with a fibrous wall lacking an epithelial lining, surrounded by chronic inflammatory infiltrate and containing granular debris (Figure 4). Features were consistent with a degenerating and calcified cysticercus, suggestive of NCC in the late granular-nodular to early nodular-calcified stage according to Escobar’s classification. Focal ghost structures and calcifications were also noted. The postoperative course was uneventful, and the patient was discharged in good condition. 

## Discussion

Diagnosing NCC can be particularly difficult when it presents as a solitary parenchymal lesion, especially in patients without obvious epidemiological risk factors. In such atypical cases, like our patient living in a non-endemic region, clinical suspicion may be low, imaging can be inconclusive, and serological tests often yield false negatives. This underscores the importance of considering NCC in the differential diagnosis of new-onset seizures, even in regions where the disease is uncommon.

Our patient’s presentation with a first-time generalized seizure is consistent with global patterns in solitary NCC, where seizures are the most frequent symptom [2,3]. Similar to our findings, a study from Eastern India found that solitary ring-enhancing lesions in the parietal lobe were the most common imaging presentation, with generalized seizures being the most frequent clinical manifestation [4]. Moreover, focal brain involvement with perilesional edema is often misinterpreted radiologically as a neoplasm or abscess, especially in non-endemic settings [5,6].

One of the most striking challenges in solitary NCC is the limited utility of serological tests. In our case, all serologic and inflammatory markers were negative. This aligns with literature reporting sensitivities as low as 50% in single-lesion disease, making serology unreliable as a sole diagnostic tool in such cases [7,8].

Harrington et al. emphasized that traditional serological methods are far more sensitive in multiple parenchymal lesions than in solitary ones, where false negatives are common [3]. This was also noted in a Japanese study, where a solitary lesion was initially misdiagnosed as a brain abscess due to negative serologies and was only confirmed postoperatively through histopathology [9,10].

Histopathological confirmation remains the diagnostic gold standard in atypical presentations. Our case revealed a degenerating cysticercus in the late granular-nodular to early nodular-calcified stage per Escobar’s classification, which is consistent with reports of solitary NCCs often being identified at transitional or calcified stages [11]. Similar histological patterns characterized by fibrotic walls, inflammatory reaction, and calcification have been reported in cases from Thailand, Japan, and Denmark, all supporting the diagnostic reliance on tissue analysis when imaging and serology are inconclusive [11-13].

Interestingly, our patient had no known route of transmission. While most NCC cases in endemic areas are linked to consumption of contaminated food or poor hygiene, sporadic cases in non-endemic countries have also been documented in patients with no travel history or apparent risk factors [14]. These cases suggest possible asymptomatic exposure during early life or undetected carriers within household environments.

The decision to proceed with surgical excision was based on diagnostic uncertainty and the presence of a solitary, calcified lesion with mass effect. While surgery is no longer routinely recommended in most NCC cases, it remains a justified approach in solitary lesions with unclear etiology, refractory seizures, or radiological suspicion of neoplasm [15]. Dhamne et al. emphasized that although medical management is preferred, surgery retains a crucial role in selected cases where imaging and serology are ambiguous and histopathological confirmation is necessary [15].

Compared to other reported cases, our management approach aligns with the growing recognition of surgery as both a diagnostic and therapeutic option in solitary NCC. Yingchoncharoen et al. similarly documented a case of solitary parenchymal NCC surgically resected after inconclusive imaging and serology, with histology confirming the diagnosis [16]. This reinforces the principle that NCC should remain in the differential diagnosis for any solitary intracranial lesion in patients from endemic regions, even in the absence of classical features or positive serology.

## Conclusions

The challenge in the cases of solitary NCC lies predominantly in the diagnosis, and when to have a high suspicion index, as the treatment course after that is simple and readily available in most institutions. It is more challenging in non-endemic areas such as in Saudi Arabia. After facing such a case, putting NCC among the differential diagnosis of solitary brain lesions is of value, as it can help change the complete course of management. NCC should be suspected in patients from endemic regions who present with seizures or other unexplained neurologic symptoms, particularly when imaging reveals cystic or contrast-enhancing lesions.
